# Production benefits on encoding are modulated by language experience: Less experience may help

**DOI:** 10.3758/s13421-023-01510-7

**Published:** 2024-04-15

**Authors:** Rachel M. Brown, Tanja C. Roembke

**Affiliations:** 1 Biological and Social Psychology, Institute of Psychology, RWTH Aachen University, Dennewartstrasse 25-27, 4th floor, room B4.25, D-52068 Aachen, Germany; 2 Cognitive and Experimental Psychology, Institute of Psychology, RWTH Aachen University, Aachen, Germany

**Keywords:** Production effect, Bilingualism, Encoding, Sensorimotor integration

## Abstract

Several lines of research have shown that performing movements while learning new information aids later retention of that information, compared to learning by perception alone. For instance, articulated words are more accurately remembered than words that are silently read (the *production effect*). A candidate mechanism for this movement-enhanced encoding, sensorimotor prediction, assumes that acquired sensorimotor associations enable movements to prime associated percepts and hence improve encoding. Yet it is still unknown how the extent of prior sensorimotor experience influences the benefits of movement on encoding. The current study addressed this question by examining whether the production effect is modified by prior language experience. Does the production effect reduce or persist in a second language (L2) compared to a first language (L1)? Two groups of unbalanced bilinguals, German (L1) – English (L2) bilinguals (Experiment 1) and English (L1) – German (L2) bilinguals (Experiment 2), learned lists of German and English words by reading the words silently or reading the words aloud, and they subsequently performed recognition tests. Both groups showed a pronounced production effect (higher recognition accuracy for spoken compared to silently read words) in the first and second languages. Surprisingly, the production effect was greater in the *second* languages compared to the first languages, across both bilingual groups. We discuss interpretations based on increased phonological encoding, increased effort or attention, or both, when reading aloud in a second language.

## Introduction

We constantly seek out new information when reading articles, making financial decisions, or following a course of study. How can we better retain new information, and are there specific conditions or strategies we can utilize? While we can readily learn by perceiving information, by reading the newspaper or listening to a lecture, we may improve our memory through *movement*, such as repeating a phone number aloud, taking notes during a lecture, or drawing (Allen et al., 2020; Damsgaard et al., 2020; Fernandes et al., 2018; MacLeod et al., 2010). The mechanisms underlying these learning effects are not yet well understood. One candidate mechanism may be motor-to-sensory predictions: performing movements is thought to elicit predictions of resulting sensory feedback (Mathias et al., 2021; Miall & Wolpert, 1996). Because accurate prediction depends on prior learning (Bar, 2007; Bubic et al., 2010), the benefit of movement on encoding may also depend on experience. When action-perception associations are in place, performing actions should prime associated percepts and may thereby aid their retention. Building on this notion, the question arises whether more extensive experience acquiring action-perception associations strengthens this cross-modal priming and thereby increases the benefit of movement on encoding. The current study examined this prediction.

Several lines of research suggest that performing movements while learning particular items improves retention of those items, compared to perceptual learning alone. One such robust phenomenon in the language domain, known as the *production effect*, occurs when participants first study lists of words by speaking them out loud or silently reading (or hearing) them, and subsequently show increased recognition accuracy for the spoken words compared to the silently studied words (Forrin et al., 2012; MacLeod et al., 2010; Mama & Icht, 2016; Murray, 1965; Ozubko et al., 2012). Notably, other types of production (i.e., movement) such as writing, typing, whispering, and gesturing, also appear to benefit word recognition compared to perceptual learning conditions involving either reading or hearing words (Forrin et al., 2012; Mama & Icht, 2016; Mathias et al., 2021). Movement has also been associated with memory enhancements in other contexts, involving different types of movements when learning different types of items. For instance, sets of instructions are better recalled after having acted or performed those instructions compared to having only heard or read the instructions (the enactment effect; Allen et al., 2020; Makri & Jarrold, 2021). In addition, drawing appears to benefit later word recognition and recall, compared to viewing or imagining word referents at learning (Fernandes et al., 2018; Wammes et al., 2019). In the studies described above, memory is typically tested in unimodal conditions that do not involve production, such as visual or auditory recognition. Overall this evidence points to a possible multi-modal encoding benefit: encoding may improve when more input (or output) channels are engaged (Mama & Icht, 2016; Wammes et al., 2019). Interestingly, recent evidence suggests that movement may confer a unique benefit to encoding. When particular component processes involved in drawing were isolated and compared, movement appeared to contribute relatively more to word memory than viewing or imagining the word referents. While word recognition was greatest after drawing, drawing blindly or simply tracing the outline of an image also increased recognition accuracy more than viewing or imagining images (Wammes et al., 2019). Thus, movement may make a unique contribution to multi-modal encoding.

The question remains: by what mechanisms could movement improve memory? Applying frameworks for action (movement) – perception coupling – one can assume that performing a movement should activate the corresponding percept that normally results from that movement, via motor-to-sensory predictions (Mathias et al., 2021; Miall & Wolpert, 1996). Reciprocal action-perception associations should additionally allow percepts, such as words or objects, to activate corresponding motor associations (Hommel, 2009; Koch et al., 2004; Pfister, 2019; Shin et al., 2010). Thus, movements are assumed to be tightly and reciprocally coupled to their corresponding percepts, which may help explain the effect of movement on memory. In accord with this idea, recent evidence suggests that motor regions may contribute directly to memory for items learned with movement. Participants learned novel words and their translations into their first language, with or without performing gestures that corresponded to the translations. In later memory tests, transcranial magnetic stimulation (TMS) over primary motor cortex slowed the recognition of novel word translations learned with gestures, compared to novel words learned without gestures (Mathias et al., 2021). The motor system may contribute to encoding via motor-to-sensory predictions (forward models), which may improve encoding and later retention.

From the viewpoint of a sensorimotor prediction hypothesis for movement-enhanced encoding, a necessary pre-condition for this hypothesis is that accurate predictions (for instance, accurate internal models) have been established through prior experience (Bar, 2007; Bubic et al., 2010; Guenther, 2006; Mathias et al., 2021; Miall & Wolpert, 1996). Forward models for example are assumed to be improved by learning movement-feedback contingencies, which then enable accurate motor-to-sensory predictions (Miall & Wolpert, 1996). Evidence from the music domain suggests that skilled performers with well-established audio-motor associations can use movement at encoding to enhance later recognition. Musicians showed greater recognition accuracy for melodies they had performed compared to those they had only listened to during learning (R. M. Brown & Palmer, 2012; Mathias et al., 2014). Additionally, musicians showed greater cortical responses to deviations within melodies they had performed at learning, compared to melodies they had only heard at learning (Mathias et al., 2014). Similarly, non-musicians who had practiced performing a musical sequence for 2 weeks showed greater cortical responses to sequence deviations compared to non-musicians who had learned the sequence by listening only (Lappe et al., 2008). These results suggest that when sensorimotor associations are established through experience, performing at learning may improve sensorimotor predictions, which may later aid recognition. The question is, could sensorimotor prediction help explain the production effect in the language domain? That is, do the acquired phoneme-motor associations of one’s native language play a role in the production effect?

A key question that has not yet been addressed is how much prior experience is needed for movement to benefit encoding. When participants have shown movement-related memory improvements, it could be assumed that they had prior experience to draw upon. The production effect has been demonstrated when participants studied words in their native language (Forrin et al., 2012; Kaushanskaya & Yoo, 2011; MacLeod et al., 2010; Ozubko & MacLeod, 2010; Zamuner et al., 2016), and when they learned non-words (and their referents) that followed the phonological rules of their native language (Kaushanskaya & Yoo, 2011; MacLeod et al., 2022). Notably, the production effect was not detected when participants learned non-words with an unfamiliar phonology (Kaushanskaya & Yoo, 2011). These findings raise the possibility that the production effect may rely on, or improve with, prior sensorimotor training, such as well-established phoneme-motor associations in one’s native language. The question then arises: how much experience may be necessary for movement to benefit memory? If sensorimotor prediction plays a role in movement-enhanced encoding, does the memory benefit increase with greater amounts of prior experience? In other words, does the production effect increase in one’s native language and decrease in a second language?

The current study examined the role of prior experience on the memory benefits of movement by examining the production effect in the context of bilingualism. Specifically, we examined whether the production effect increased when bilinguals encoded and recognized words in their first language compared to words in their second language. Importantly, we focused on unbalanced bilinguals whose experience in their first language was relatively greater than that of their second language. In two experiments, two independent groups of unbalanced bilinguals, German-English bilinguals (Experiment 1) and English-German bilinguals (Experiment 2), studied lists of German or English words (learning tasks) and later recognized words from the lists. Each participant performed a learning task followed by a recognition task twice: once with only German words and once with only English words. During learning, participants were presented with words on a screen one at a time, and they either read them silently or read them while also speaking them out loud. In a subsequent recognition task, participants were presented with new and old words, and responded “yes” or “no” according to whether they recognized each word or not. We predicted higher recognition accuracy for words that were spoken compared to those that were silently read (a production effect). Crucially, we also predicted a larger difference between spoken and silently read words in participants’ first language (L1) compared to their second language (L2), on the premise that the production effect may be influenced by sensorimotor predictions that are experience-dependent. Note that we here assume that “experience” can include the influence of both time (amount of exposure or practice) and age (sensitive periods for language acquisition): a second language may be practiced less and learned at a later age compared to a first language.

## Experiment 1

### Method

#### Participants

A sample size of *N* = 57 per experiment was estimated from an a priori power analysis. Because the forward model perspective does not make explicit assumptions about effect sizes, we instead estimated an effect size of *η*^*2*^_*p*_ = .16 based primarily on a reported interaction between spoken versus silent encoding of linguistic items (non-words) and the familiarity of the material (familiar vs. unfamiliar phonology) (Kaushanskaya & Yoo, 2011), similar to the planned analysis for the current study. Power analysis using an alpha level of 0.05 and a power level of 0.80 suggested a sample size of *N* = 57. To verify participants’ knowledge of German and English we administered the LexTALE word identification task in each language (Lemhöfer & Broersma, 2012) in addition to a language background questionnaire (see below).

Sixty-six German-English bilingual volunteers from the RWTH Aachen University student community participated in the experiment and received course credits as compensation. Eight participants were excluded: two reported that they were not native German speakers, one did not confirm that they spoke English, one performed below a pre-determined 75% accuracy cutoff on the German LexTALE task, one performed below a pre-determined 50% accuracy cutoff on the English LexTALE task, and three performed more than 5% of the experiment trials incorrectly (incorrectly speaking or silently reading on a given trial). The final sample consisted of 58 participants, all of whom reported to be native German speakers with English as a second language (note that many participants spoke multiple languages, see Table 1), and they reported having normal or corrected-to-normal vision and to being neurologically healthy. The sample included 51 females and 53 right-handed participants with a mean age of 21.3 years (*SD* = 3.73, range 18–37 years). See Table 1 (German L1) for a summary of participants’ language backgrounds. Participants provided informed consent, and the study procedures were conducted according to the 1964 Declaration of Helsinki and its later amendments.

**Table 1:** Language background characteristics in Experiment 1 and Experiment 2

	German L1	English L1
	*M (SD)*	*M (SD)*
L2 Age of acquisition	8.2 (2)	9.5 (8)
L2 Years of study	10.2 (2)	10.4 (8)
L2 Self-rated Abilities		
Speaking	5.3 (1.0)	5.3 (1.4)
Understanding	5.8 (0.8)	5.7 (1.2)
Reading	5.6 (0.9)	5.5 (1.3)
Writing	4.9 (1.0)	4.8 (1.5)
LexTALE		
L1	87% (4.5)	92% (6.7)
L2	73% (9.1)	69% (10.5)
Other Languages	1.4 (0.8)	0.8 (0.9)
	*n*	*n*
Other Languages (by number)		
No other languages	8	29
One other language	24	15
Two other languages	22	11
Three other languages	4	2
L2 Self-reported Levels		
A2 (beginner)	1	6
B1 (intermediate)	5	12
B2 (advanced)	40	19
C1 (expert)	12	20

German L1 = German-English bilinguals, Experiment 1, English L1 = English-German bilinguals, Experiment 2, L2 Age of acquisition = the age at which participants reported having started to learn the L2, L2 Years of study = the number of years participants reported having studied the L2, L2 Self-rated Abilities = self-rated abilities on a scale of 1 to 7 (1 = not at all, 7 = fluent), Other Languages = the number of other languages participants reported speaking, LexTALE = percent correct performance on the LexTALE lexical decision task, *M* = mean, *SD* = standard deviation

#### Materials

Stimuli consisted of words that were drawn from a pool of 120 English nouns and 120 German nouns. The English pool was the same 120 words listed in the Appendix of MacDonald and MacLeod (MacDonald & MacLeod, 1998) and later used in the experiments reported by MacLeod and colleagues (MacLeod et al., 2010). English nouns were five to ten letters long and had frequencies greater than 30 per million (see MacLeod et al., 2010). The German words were selected from the SUBTLEX-DE database (Brysbaert et al., 2011) such that they matched the English words in length and in mean log frequency, as recommended by Brysbaert and colleagues (Brysbaert et al., 2011). The words presented to each participant in each language were drawn randomly from each pool (see below).^1^ In addition, items for a practice task consisted of the German words for the numbers 1 through 10.

#### Procedure

The experiment was conducted online using Gorilla (gorilla.sc). Participants used their own computers to complete the study and were required to use a laptop or a desktop with a microphone. All instructions were presented in German, except for the instructions for the English LexTALE and the instructions for the English learning and recognition tasks. Before beginning the experiment, participants were asked to test their microphone by making a short recording of their voice and playing it back. Participants were asked to exit the experiment if the recording did not work. All vocal responses during the experiment were recorded by the participant’s microphone.

Participants first completed two word-identification tasks (LexTALE task), the first one in German and the second one in English. On each trial participants were presented with a string of letters, and they were instructed to decide whether it was an existing word in the respective language by pressing the “J” key if they thought it was an existing word (even if they did not know its meaning) and the “K” key if they thought it was not an existing word (or were not sure). Participants were given 5 seconds (s) to respond on each trial. Each task included 60 trials, half of which were words and half of which were non-words, presented in a pseudorandom order.

Participants then completed a brief practice task in which ten German number words were presented one at a time in either blue or white, in a pseudorandom order, against a grey background. Participants were instructed to speak aloud the words presented in blue, and to silently read the words presented in white. Each trial began with a blank screen shown for 500 milliseconds (ms), followed by the presentation of a word in either blue or white in the center of the screen, at which point the microphone began recording. Each word remained on-screen for 2 s, after which the recording stopped and the next trial began automatically. The aim of the practice task was to ensure that participants understood when to speak aloud and when to remain silent.

The main part of the experiment consisted of a learning task followed by a recognition task, both of which were completed once with only English words and once with only German words, the order of which was counter-balanced across participants. In other words, each participant completed the learning and recognition task first in one language, and then completed the learning and recognition task again in the other language. The procedure of the learning and recognition tasks was modelled closely after MacLeod and colleagues (MacLeod et al., 2010) in order to replicate the classical production effect and to facilitate comparison between the present and previous results. For each participant and for each language (German and English), 80 words were randomly selected from the 120-word pool. Each of those 80 words were then randomly divided into two sets of 40 words, such that 40 words were presented in blue and 40 words were presented in white during the learning task, in a random order. In addition, 20 words were then randomly selected from each set of 40 words, and an additional 20 words were randomly selected from the remaining 40 words that were not presented at learning. These 60 words were presented in yellow during the recognition task.

During the learning task, each trial began with a blank screen presented for 500 ms, followed by the presentation of a blue or white word against a grey background in the center of the screen, at which point the microphone began recording. Each word remained on-screen for 2 s, after which the recording stopped and the next trial began automatically. The learning task consisted of 80 trials. The recognition task began immediately after the learning task, after a short set of instructions. During the recognition task, each trial began with a blank screen presented for 500 ms, followed by the presentation of a yellow word against a grey background in the upper part of the screen and two response buttons, one labelled “Ja” or “Yes” and one labelled “Nein” or “No” (for the German or English recognition task, respectively), in the lower part of the screen. The word remained on-screen until the participant clicked with their mouse on one of the response buttons, after which the next trial began immediately. The recognition task consisted of 60 trials.

Participants ended the experiment by answering questions related to their language background and demographic information, and they were asked to verify whether they were neurologically healthy. The entire experiment lasted approximately 30 min.

Audio recordings from each learning task trial for each participant were coded offline by hand according to whether participants correctly remained silent or spoke aloud according to the instructions presented during the task. For any trial that did not comply with the instructions, the corresponding item for that participant was discarded from further analysis.^2^

### Results

#### Recognition

**Fig. 1:**
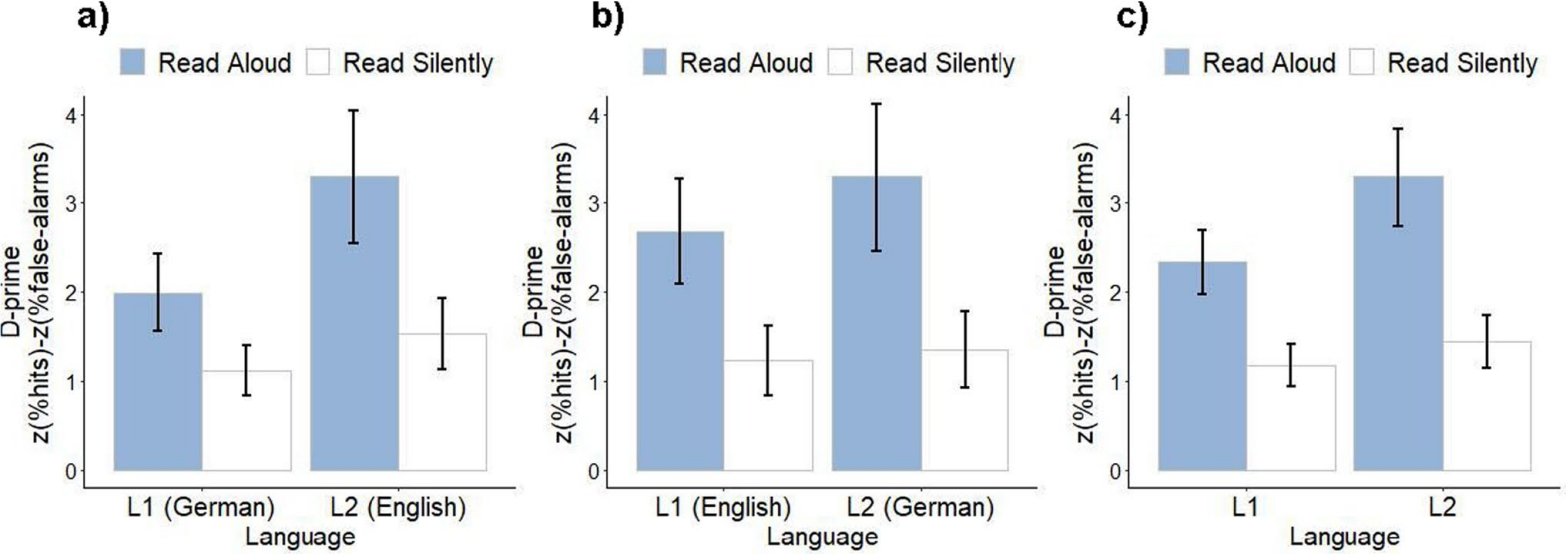
Production by language: Experiment 1, Experiment 2, and Combined. *Note*. Effects of Production (Read Aloud = Spoken, vs. Read Silently = Silent) and Language (L1 vs. L2) on recognition accuracy (d-prime scores) for each language group, and across groups. (**a**) German – English bilinguals (Experiment 1). (**b**) English – German bilinguals (Experiment 2). (**c**) Both groups combined (L1 = German or English, L2 = German or English). In all panels (**a**, **b**, and **c**) marginal means are plotted and error bars represent 95% confidence intervals

Recognition accuracy was assessed by calculating both hit rates as well as d-prime scores (hit rate minus false alarm rate, both z-normalized) in order to account for possible response bias, similar to previously reported procedures (Fernandes et al., 2018; Wammes et al., 2019). Hit rates and false alarm rates are reported in Table 2. A 2 (Production: Spoken vs. Silent) × 2 (Language: L1 (German) vs. L2 (English)) within-subjects ANOVA on hit rates showed main effects of Production (Spoken > Silent, *F*(1, 57) = 203.99, *p* < .001, *η*^*2*^_*G*_ = .418 , *η*^*2*^_*p*_ = .78) and Language (English (L2) > German (L1), *F*(1, 57) = 18.63, *p* < .001, *η*^*2*^_*G*_ = .043 , *η*^*2*^_*p*_ = .25), and no interaction (*F*(1, 57) = 2.02, *p* = .161, *η*^*2*^_*G*_ = .005, *η*^*2*^_*p*_ = .03). A second 2 × 2 within-subjects ANOVA (with the same fixed factor structure) on d-prime scores showed main effects of Production (Spoken > Silent, *F*(1, 57) = 95.70, *p* < .001, *η*^*2*^_*G*_ = .187, *η*^*2*^_*p*_ = .63) and Language (English (L2) > German (L1), *F*(1, 57) = 21.19, *p* < .001, *η*^*2*^_*G*_ = .089, *η*^*2*^_*p*_ = .27). In addition, a Production by Language interaction was shown (*F*(1, 57) = 14.86, *p* < .001, *η*^*2*^_*G*_ = .025, *η*^*2*^_*p*_ = .21) such that recognition accuracy was relatively greater when speaking English (L2) words aloud compared to speaking German (L1) words aloud (*Spoken English*: *M* = 3.28, *SE* = 0.275, 95%CI = [2.733 3.83], *Spoken German*: *M* = 1.99, *SE* = 0.157, 95%CI = [1.672 2.30], *difference* = 1.296, *SE* = 0.263), *t* = 4.926, *p* < .0001, *Hedge’s g* = .72). The difference between recognition accuracy for English and German words that were silently read was relatively smaller (*Silent English*: *M* = 1.53, *SE* = 0.146, 95%CI = [1.236 1.82], *Silent German*: *M* = 1.12, *SE* = 0.101, 95%CI = [0.912 1.32], *difference* = 0.413, *SE* = 0.161, *t* = 2.562, *p* = .0131, *Hedge’s g* = .42) (see Fig. 1a).

**Table 2:** Percentage of “Yes” responses at recognition: Experiment 1

	Spoken aloud			Silent			Not studied		
	*M*	*SE*	*CI*	*M*	*SE*	*CI*	*M*	*SE*	*CI*
German (L1)	83.0	1.35	[80.3 85.7]	61.8	2.05	[57.7 65.9]	25.2	1.70	[21.8 28.6]
English (L2)	90.6	1.12	[88.4 92.9]	65.7	2.39	[60.9 70.5]	22.3	1.96	[18.4 26.2]

CI = 95% confidence intervals

### Discussion

Experiment 1 showed a robust production effect in both a first and a second language. In addition, we observed that recognition accuracy (as measured by d-prime scores) was greater after speaking words aloud in a second language (English) compared to speaking words aloud in a first language (German). This result suggests a greater production effect in a second language compared to a first language, contrary to our initial prediction.

In order to generalize this finding both to a different bilingual group and to a larger and more diverse population (not limited to a German university), a second experiment presented the same task and stimuli to unbalanced English-German bilinguals recruited via Prolific, an online recruiting service available to people around the world. Based on the findings of Experiment 1, we predicted that English-German bilinguals would show a greater production effect for German words (a second language) compared to English words (their first language).

## Experiment 2

### Method

#### Participants

Eighty English-German bilingual volunteers participated in the study via Prolific (prolific.co) and received monetary compensation (£3.75 total, approximately £7.5/h). Pre-screening criteria limited online recruitment to individuals who had upon registration with Prolific declared themselves to be between the ages of 18 to 35 years, to have normal or corrected-to-normal vision, and to be both native English speakers and fluent in German. After completing the study, participants were asked to report their age, vision correction, and if they were neurologically healthy. A total of 23 participants were excluded (29%) based on pre-determined criteria: one participant did not follow the task instructions on more than 5% of the trials, nine participants did not report that they were neurologically healthy, five participants reported that they were not native English speakers, seven participants scored below 50% on the German LexTALE, and one participant scored well below 75% on the English LexTALE (63%). Because of the high attrition rate, one participant was included who scored very close to the English LexTALE cutoff (73%) and met all other eligibility criteria. This yielded a final sample of *N* = 57, all of whom reported that they were native English speakers with German as a second language (note that some participants spoke multiple languages, see Table 1), and they reported having normal or corrected-to-normal vision and to be neurologically healthy. The sample included 35 females and 49 right-handed participants with a mean age of 26.4 years (*SD* = 4.88, range 19–35 years). See Table 1 (English L1) for a summary of participants’ language background. Participants provided informed consent, and the study procedures were conducted according to the 1964 Declaration of Helsinki and its later amendments.

#### Materials

The same set of 120 English and 120 German nouns were used. For the practice task, the English words for the numbers 1 through 10 were presented.

#### Procedure

The experiment was conducted online using Gorilla. All aspects of the procedure were identical to those of Experiment 1, with the following exceptions. All instructions were presented in English, except for the instructions for the German LexTALE and the instructions for the German learning and recognition tasks. Participants completed the English LexTALE followed by the German LexTALE.

### Results

#### Recognition

Hit rates and false alarm rates are reported in Table 3. A 2 (Production: Spoken vs. Silent) × 2 (Language: L1 (English) vs. L2 (German)) within-subjects ANOVA on hit rates showed main effects of Production (Spoken > Silent, *F*(1, 56) = 313.35, *p* < .001, *η*^*2*^_*G*_ = .558 , *η*^*2*^_*p*_ = .85) and Language (German (L2) > English (L1), *F*(1, 56) = 4.88, *p* = .031, *η*^*2*^_*G*_ = .012 , *η*^*2*^_*p*_ = .08), and no interaction (*F*(1, 56) = 0.12, *p* = .735, *η*^*2*^_*G*_ < .001 , *η*^*2*^_*p*_ = .002). A second 2 × 2 within-subjects ANOVA (with the same fixed factor structure) on d-prime scores showed a main effect of Production (Spoken > Silent, *F*(1, 56) = 112.07, *p* < .001, *η*^*2*^_*G*_ = .217, *η*^*2*^_*p*_ = .67), and no main effect of Language (*F*(1, 56) = 2.22, *p* = .141, *η*^*2*^_*G*_ = .013, *η*^*2*^_*p*_ = .04). A near-significant Production by Language interaction was shown (*F*(1, 56) = 3.74, *p* = .058, *η*^*2*^_*G*_ = .006, *η*^*2*^_*p*_ = .06), such that recognition accuracy was marginally greater when speaking aloud in German compared to speaking aloud in English (*Spoken German*: *M* = 3.28, *SE* = 0.305, 95%CI = [2.671 3.89], *Spoken English*: *M* = 2.67, *SE* = 0.216, 95%CI = [2.239 3.10], *difference* = 0.611, *SE* = 0.339), *t* = 1.804, *p* = .0766, *Hedge’s g* = .30), whereas there was little difference in recognition accuracy between silently reading in German versus English (*Silent German*: *M* = 1.35, *SE* = 0.158, 95%CI = [1.031 1.66], *Silent English*: *M* = 1.22, *SE* = 0.141, 95%CI = [0.942 1.51], *difference* = 0.123, *SE* = 0.195), *t* = 0.628, *p* = .5323, *Hedge’s g* = .11) (see Fig. 1b).

**Table 3:** Percentage of “Yes” responses at recognition: Experiment 2

	Spoken aloud			Silent			Not studied		
	*M*	*SE*	*CI*	*M*	*SE*	*CI*	*M*	*SE*	*CI*
English (L1)	86.3	1.38	[83.5 89.1]	55.5	2.24	[51.0 60.0]	21.6	2.06	[17.5 25.7]
German (L2)	89.7	1.21	[87.3 92.1]	58.2	2.33	[53.5 62.8]	19.5	1.79	[15.9 23.1]

CI = 95% confidence interval

#### Comparison of Experiments 1 and 2

In order to assess the production effect in a first versus a second language independently of language, we directly compared the two language groups. A 2 (Production: Spoken vs. Silent) × 2 (Language: L1 vs. L2) × 2 (Group: native German speakers vs. native English speakers) mixed ANOVA on hit rates showed main effects of Production (Spoken > Silent, *F*(1, 113) = 516.00, *p* < .001, *η*^*2*^_*G*_ = .493 , *η*^*2*^_*p*_ = .82) and Language (L2 > L1, *F*(1, 113) = 21.13, *p* < .001, *η*^*2*^_*G*_ = .025, *η*^*2*^_*p*_ = .16), and a Production by Group interaction (*F*(1, 113) = 11.57, *p* < .001, *η*^*2*^_*G*_ = .021, *η*^*2*^_*p*_ = .09) such that the hit rate for silently read words was greater for the German speakers (*M* = 63.7, *SE* = 1.98, 95%CI = [59.8 67.7]) than for the English speakers (*M* = 56.8, *SE* = 2.00, 95%CI = [52.9 60.8]) (*difference* = 6.91, *SE* = 2.81, *t*(113) = 2.456, *p* = .0156, *Hedge’s g* = .46). The Production by Language interaction did not reach significance (*F*(1, 113) = 1.69, *p* = .197, *η*^*2*^_*G*_ = .002, *η*^*2*^_*p*_ = .01). A second 2 × 2 × 2 mixed ANOVA (with the same fixed factor structure) on d-prime scores showed main effects of Production (Spoken > Silent, *F*(1, 113) = 207.90, *p* < .001, *η*^*2*^_*G*_ = .202, *η*^*2*^_*p*_ = .65) and Language (L2 > L1, *F*(1, 113) = 15.77, *p* < .001, *η*^*2*^_*G*_ = .04, *η*^*2*^_*p*_ = .12). In addition, a Production by Language interaction was shown (*F*(1, 113) = 16.21, *p* < .001, *η*^*2*^_*G*_ = .013, *η*^*2*^_*p*_ = .13), such that recognition accuracy was relatively greater after speaking aloud in one’s L2 compared to speaking aloud in one’s L1 (*Spoken L2*: *M* = 3.28, *SE* = .2051, 95%CI = [2.876 3.69], *Spoken L1*: *M* = 2.33, *SE* = .1331, 95%CI = [2.065 2.59], *difference* = 0.964, *SE* = 0.214), *t* = 4.455, *p* < .0001, *Hedge’s g* = .50), compared to silently reading in one’s L2 versus L1 (*Silent L2*: *M* = 1.44, *SE* = .1073, 95%CI = [1.225 1.65], *Silent L1*: *M* = 1.17, *SE* = .0867, 95%CI = [0.998 1.34], *difference* = 0.268, *SE* = 0.126, *t* = 2.117, *p* = .0364, *Hedge’s g* = .26) (see Fig. 1c). These results were additionally replicated using a generalized linear mixed effects model analysis (see Appendix 1).

### Discussion

Experiment 2 showed a robust production effect in both a first and a second language, similar to Experiment 1. In addition, we observed that recognition accuracy (as measured by d-prime scores) was marginally greater after speaking words aloud in a second language (German) compared to doing so in a first language (English), in an independent group of unbalanced bilinguals. Thus, the English-German bilinguals showed a similar, though less pronounced, L2 advantage for production. The less-pronounced L2 production effect may have been due to greater L2 background variability (note, e.g., the standard deviations in Table 1) or to other sources of variability that were present due to sampling from a larger population. A direct comparison of the two language groups suggested a consistent pattern across the two groups: speaking aloud yielded greater recognition accuracy (d-prime) in a second language (German or English) compared to a first language (German or English). Additionally, hit rates for all silently read words were higher for the native German speakers compared to the native English speakers, possibly due to different response biases between the two groups.

## General discussion

### Summary of findings

We observed evidence that the production effect may be greater in a second language compared to a first language. Bilinguals showed higher recognition accuracy for words they had spoken compared to words they had silently read, replicating the production effect. Furthermore, recognition accuracy for spoken words was greater in a second language (L2) compared to a first language (L1). This pattern was generally consistent across two language groups: German-English bilinguals and English-German bilinguals (all performing the same task with the same stimuli). Below we consider how domain-specific (i.e., psycholinguistic) and domain-general processes could contribute to the findings.

### Psycholinguistic processes

One way to interpret the results is to consider whether phonological processing plays a role in the production effect. Reading in a second language may have involved transforming written words (orthographic form) into the corresponding phonemes (sounds) and then into meaning (semantics), while reading in a first language may have involved associating written words directly with their meaning (Coltheart et al., 2001; Jobard et al., 2003; Seidenberg, 2005; Seidenberg & McClelland, 1989). Some evidence suggests that sound-meaning associations may be utilized less with greater reading experience (Wise Younger et al., 2017). In early childhood, phonological abilities and activation in regions implicated in phonological processing (e.g., inferior parietal regions) were associated with later orthographic abilities (Sprenger-Charolles et al., 2003) and reading improvements (Wise Younger et al., 2017). Adults learning a second language also showed inferior parietal activation, which additionally correlated with proficiency gains (Barbeau et al., 2017). Unbalanced bilinguals showed greater activation in brain regions involved in articulation and orthographic-phonological associations (inferior frontal gyrus, premotor cortex, fusiform gyrus) when reading out loud in their second language compared to their first language (Berken et al., 2015). If reading in a second language relies more on phonological processing than in a first language, articulating L2 words might prime phonological associations, which may increase the production effect. However, from this perspective one could also assume that articulation is redundant to phonological processing in a second language and thus may not offer a novel encoding process.

Contrary to the notion that phonological processing may decrease when reading in a first language, some evidence suggests that skilled readers are influenced by phonological features of words (Van Orden, 1987). If we assume then that reading in a first language could utilize phonological processing to an equal or even greater extent than a second language, we could suppose that articulation offers a unique or non-redundant form of processing to L2 reading that in turn benefits encoding. Thus, remaining questions include: (1) the degree to which a second language relies on phonological processing, (2) whether articulation complements or primes phonological codes, and (3) whether phonology contributes to the production effect. To date there is some evidence to support a role of phonological processing in the production effect. An fMRI study showed that speaking words aloud at encoding increased response in both somatosensory and auditory regions (e.g., planum temporale) implicated in phonological processing, compared to either silently reading or speaking aloud a control word (“check”), and these regions correlated with improved recollection (Bailey et al., 2021). At the same time, a recent study found little to no modulation of the production effect when phonologically similar distractors were present at recognition (Fawcett et al., 2022). Further work will be needed to answer these questions.

### Domain-general processes

Another way to interpret the results is to consider the role of domain-general processes, for example the cognitive control requirements of articulating in one’s first versus second language. One possibility is that reading or remembering words in a second language is less demanding compared to a first language. Some research suggests that recognition accuracy is higher for words in a second language compared to a first language (Durgunoǧlu & Roediger, 1987; Francis et al., 2018; Francis & Gutiérrez, 2012), which we replicated as main effects in the current study. It was proposed that L2 words may be less susceptible to contextual or semantic competition during memory retrieval. A similar interpretation has been used to explain superior recognition of low-frequency compared to high-frequency words (Francis & Strobach, 2013). If we apply this interpretation to the current findings, we still need to explain the increased production effect in a second language, thus an additional mnemonic process would need to be assumed.

Additionally, speaking out loud in a second language may be more demanding than speaking out loud in a first language, which may lead to a memory advantage. For instance, the desirable difficulty perspective posits that learning outcomes should improve when greater cognitive effort is applied at encoding, provided that the learning task is not overly challenging (Bjork & Bjork, 2019, 2020; Eskenazi & Nix, 2021). Consistent with this idea, the production effect was amplified when participants spoke words out loud following a short retention delay after reading the words (Mama & Icht, 2018). Delayed vocal production at encoding resulted in greater recall accuracy compared to both silent reading and immediate vocal production at encoding. Additionally, delayed vocal production resulted in greater recall accuracy when the word was presented only once before delayed production, compared to when the word was presented again (unpredictably) at production. The authors attributed recall improvements to the increased difficulty of encoding items by both reading and maintaining items in working memory, compared to only reading (Mama & Icht, 2018). According to this perspective, speaking in a second language may have improved recognition memory due to its difficulty relative to speaking in a first language or reading silently in a second language. Further work is needed to clarify the processes (e.g., arousal) that contribute to memory-enhancing difficulty.

One possibility is that increased attention to L2 words at encoding improved later retention, particularly when they were spoken. For example, word recall improved when participants made more errors while pronouncing words at encoding, which may have led to increased attentional monitoring (Phaf & Wolters, 1993). Other work suggested that the production effect may decrease in the presence of distraction: the production effect failed to be detected when amplitude-fluctuating background noise or background speech was presented during encoding by reading aloud or silently, but the effect remained in the presence of steady-state background noise (Mama et al., 2018). In the current study, it could be that L2 words increased attention when they were spoken, compared to silently reading them or compared to speaking L1 words. Further work would be required to clarify when and how attention is engaged during the process of speaking in a second language. Speaking may increase the salience of L2 words, if, for example, articulation or auditory feedback captures (orients) attention. Speaking in a second language may additionally increase the monitoring of speech output (e.g., Phaf & Wolters, 1993). Additionally, attention may be engaged prior to speaking. Recent work examining electrophysiological signatures of the production effect showed a P3b response increase during encoding when participants saw the instruction to speak out loud (prior to speaking), compared to seeing the instruction to remain silent (Hassall et al., 2016; Zhang et al., 2023) or to say a control word (“check”) (Zhang et al., 2023). The authors suggested that speech preparation, involving attention or other preparatory processes, may play a role in the production effect (Zhang et al., 2023). It would be interesting to see if these findings extend to a second language.

Based on the available evidence, we suggest that both psycholinguistic and domain-general processes provide plausible explanations for the current results and both merit further investigation. The influence of bilingualism on the production effect raises further questions about the role of language production processes in memory. Neuroimaging evidence is consistent with a role for phonological processes in the production effect (Bailey et al., 2021) and in second-language reading (Berken et al., 2015), yet some work also calls this role into question (e.g., Fawcett et al., 2022). In addition, it is reasonable to assume that speaking in a first and second language have different cognitive demands that could in turn impact both language production and encoding. For example, phonological processing when reading in a second language could involve directing attention to phonological planning or output. A combination of attentional and phonological processes may also be helpful for explaining seemingly contradictory findings, such as a production effect increase when singing (Quinlan & Taylor, 2019), but a decrease when speaking as a popular character (Wakeham-Lewis et al., 2022). Considering multiple processes (Zhang et al., 2023) may help advance our understanding of the memory benefits of production.

### The production effect and expertise

The current study suggests that the production effect does not depend on extensive lifelong sensorimotor experience, such as the experience that underlies one’s first language. Previous work suggests that some experience is still necessary (see, e.g., Lappe et al., 2008). The production effect has been shown for novel words and novel word *referents* after training, when those novel words used native-language phonology. English speakers more accurately recognized English-sounding non-words (MacLeod et al., 2022) or their referents (English words or images) (Kaushanskaya & Yoo, 2011; Zamuner et al., 2016) when the non-words were spoken aloud compared to read silently. When non-words were phonologically unfamiliar, no increase in referent recognition after speaking aloud was detected (Kaushanskaya & Yoo, 2011). Interestingly, performing gestures helped people learn novel words with a less-familiar (non-native) phonology. Participants more accurately recognized the novel word translations they had studied while performing gestures corresponding to the referents compared to those they studied while viewing pictures of the referents (Mathias et al., 2021). Thus, while some experience may be critical for a production effect, extensive experience does not seem to be necessary. Whether there is an optimal level of experience remains to be seen.

## Limitations

It is important to note that first and second languages may be processed differently as a function of either time-dependent factors (exposure/practice) or sensitive periods for language acquisition. For example, when unbalanced bilinguals were matched on language proficiency to balanced (simultaneous) bilinguals, language processing differences were still seen between the groups (e.g., greater phonologically relevant neural activation when reading in a second language) (Berken et al., 2015). In the current study we cannot disentangle the influences of acquisition age and years of exposure or practice to the L2 production effect.

To the best of our knowledge, the current results are among the first to report a production effect outside of participants’ native language, and this potentially involves pronunciation challenges. We focused our task instructions and data inclusion on task compliance: we did not provide any specific instructions for pronunciation, and responses were included if they complied with instructions to speak or remain silent. We expected pronunciations to differ greatly across participants, which precluded adopting strict pronunciation criteria. We also reasoned that pronunciation errors should work against the production effect, as words pronounced differently might be difficult to recognize later, which would mean that our reported effect sizes may be conservative.^3^

Another limitation of this study is that we cannot draw strong conclusions about how language proficiency influences the production effect at an individual level. That is, the language background information and the LexTALE scores we collected were used only to establish that the groups as a whole were relatively unbalanced in terms of average L1 and L2 proficiency. Although the LexTALE task is often used to assess proficiency, it showed low-to-modest correlations with a standardized language assessment protocol (Puig-Mayenco et al., 2023). The authors argued that the LexTALE task should not be used as a diagnostic measure of global proficiency for a given individual. Further work using comprehensive and diagnostic language testing would be needed to examine how individual proficiency would predict a single individual’s production effect.

Finally, it should be noted that the languages examined here, German and English, share phonological similarities. The extent to which an increased L2 production effect is influenced by the L1-L2 similarity is an open question. If phonological similarity *decreases* the L2 production effect (if speaking in an L2 was too easy here, for example), then we may have *underestimated* the effect size of an L2 production effect. A valuable next step would be to examine bilinguals who speak highly dissimilar languages. In addition, many participants spoke multiple languages (see Table 1), particularly Experiment 1 participants, who commonly spoke Dutch (related to German and English), French, or Spanish (both less related to German or English). Multilingualism could potentially increase or decrease the L2 production effect, for example by highlighting differences or similarities between German and English, respectively. Both possibilities are suggested by exploratory regression analyses on individual L2 production effect scores (Appendix 2): speaking one additional language related to a decreased L2 production effect in Experiment 1, but speaking three additional languages related to an increased L2 production effect in Experiment 2 (although only two participants spoke three additional languages in Experiment 2, see Appendix 2). The potential influences of multilingualism would be an interesting topic for further study.

## Conclusion

We examined the role of prior experience (time- or age-dependent) on the encoding benefits of movement by examining the production effect in unbalanced bilinguals. We observed a greater production effect in a second language compared to a first language. Participants more accurately recognized words they had spoken aloud in their second language compared to their first language. This result was relatively consistent across two independent groups of unbalanced bilinguals (German-English and English-German bilinguals). We propose that psycholinguistic and/or domain-general processes may account for the findings. For example, reading in a second language may utilize phonological-motor associations, and/or increased effort or attention may be engaged in speaking in a second language. The findings here add further insight into the possible mechanisms behind encoding by movement (e.g., the production effect) by showing that less prior experience may amplify these memory benefits.

## Data Availability

The raw, anonymized datasets are freely available on psycharchives.org.

## Appendix 1

### Generalized linear mixed effects modelling

In order to model both participants and stimuli (words) as random effects, we performed a generalized linear mixed effects modelling analysis on the combined data from Experiments 1 and 2. Response accuracy at recognition was modelled as a discrete outcome for each word individually (a correct or incorrect response to each item, correct = 1, incorrect = 0) using generalized linear mixed-effects modelling (glmer() function in the lme4 package, version 1.1.33 (Bates et al., 2015) in R, version 4.3.1 (R Core Team, 2023). We followed the procedure recommended by Brown (Brown, 2021).

First, the factors “Production (Spoken/Silent)” and “Language (L1/L2)” were dummy coded to facilitate interpretation of interactions (Spoken = 1, Silent = 0, L1 = 0, L2 = 1) and the factor “Group” was sum-coded such that the average across the two groups was centered on zero (“German L1 – English L2” = -0.5, “English L1 – German L2” = 0.5) in order to facilitate interpretation of an interaction averaged over levels of “Group” (see Brown, 2021).

Next, a maximal model was constructed using the maximal possible random effects structure, below, including all possible random-effect intercepts and slopes. Note that slopes that were not possible to estimate were omitted: a slope across “Group” cannot be estimated for each participant (“Group” is a between-subject factor), and a slope across “Language” cannot be estimated for each word (“Language” is a between-word factor). (Note that the code “production*language*group” below also includes all main effects and 2-way interactions.)

$$\begin{array}{lll} \mathrm{maximal}\_\mathrm{model} = \mathrm{accuracy}\sim 1 + \mathrm{production} * \mathrm{language} * \mathrm{group} + \\ (1 + \mathrm{production} + \mathrm{language} |\mathrm{participant}) + \\ (1 + \mathrm{production} + \mathrm{group} |\mathrm{word})\end{array}$$

The above maximal model was submitted to the buildmer() function (“buildmer” package, version. 2.9 (Voeten, 2023) (as used in e.g., Elias & Degani, 2022)) in R, which finds the maximal feasible random effects structure that can be estimated by the data (that is capable of converging). Additionally, we forced the buildmer() function to include all possible fixed effects in the final model (saturated fixed effects), using the following code:

$$\begin{array}{l}\begin{array}{l}{\text{buildmer}}\,\left(\mathrm{accuracy }\sim 1+{\text{production}}*{\text{language}}*{\text{group}}+\right.\\ \left(1+{\text{production}}+{\text{language}}\left|{\text{participant}}\right.\right)+\\ \left(1+{\text{production}}+{\text{group}}\left|{\text{word}}\right.\right),\end{array}\\ \begin{array}{l}{\text{buildmerControl}}={\text{list}}\left(\,{\text{include}}=\sim {\text{production}}*{\text{language}}*{\text{group}}\right), \\ \left.{\text{data}}={\text{combined}},\mathrm{ family}={\text{binomial}}\right)\end{array}\end{array}$$

This yielded a final model (below) that included intercepts for participant and word and the slope for participant (see model summary in Table 4).

$$\begin{array}{lll} \mathrm{final}\_ \mathrm{model} = \mathrm{accuracy} \sim 1 + \mathrm{production} + \mathrm{language}+ \mathrm{production}: \mathrm{language}+ \\ \mathrm{group}+ \mathrm{production}: \mathrm{group}+ \mathrm{language}: \mathrm{group}+ \mathrm{production}: \mathrm{language}: \mathrm{group}+ \\ (1 + \mathrm{production} | \mathrm{participant}) + (1 |\mathrm{word}) \end{array}$$

**Table 4:** Model summary: Experiments 1 and 2 combined

Fixed effect	*Estimate*	*SE*	*z*	*P(z)*
Intercept	0.55351	0.10886	5.08485	<0.001
Production	1.18805	0.11520	10.31302	<0.001
Language	0.17702	0.10466	1.69132	0.091
Group	-0.28913	0.15354	-1.88317	0.060
Production × Language	0.54103	0.15982	3.38531	0.001
Production × Group	0.55546	0.16426	3.38152	0.001
Language × Group	-0.06327	0.16281	-0.38860	0.698
Production × Language × Group	-0.32077	0.22679	-1.41441	0.157
Random effect	*Variance*	*SD*	*Correlation*	
Word (intercept)	0.1488	0.3858		
Participant (intercept)	0.3679	0.6066		
Production	0.1162	0.3409	-0.35	

*SE* = standard error, *SD* = standard deviation

Likelihood ratio tests were then conducted on all fixed effects in the above model using the mixed() function (“afex” package in R, v. 1.3.0) (Singmann et al., 2023) using the following code:

$$\begin{array}{l}{\text{mixed}}({{\text{final}}}\_\mathrm{model},\mathrm{data }=\mathrm{ combined},\mathrm{ family }=\mathrm{ binomial}, \\ \mathrm{control }=\mathrm{ glmerControl}(\mathrm{optimizer }="{\text{bobyqa}}"),\mathrm{ method }= "{\text{LRT}}")\end{array}$$

Results showed that the Production by Language interaction contributed significantly to the model fit (see Table 5).

**Table 5:** Likelihood ratio tests: Experiments 1 and 2 combined

Effect	*df*	*Chi squared*	*p*
Production	1	221.42	<.001
Language	1	35.04	<.001
Group	1	1.03	.310
Production × Language	1	11.19	<.001
Production × Group	1	8.63	.003
Language × Group	1	2.16	.142
Production × Language × Group	1	1.97	.160

We additionally performed a generalized linear mixed effects model analysis for each Experiment separately, following the same procedure as above (without the fixed factor “Group”). The maximal model submitted to the buildmer() function (using a saturated fixed factor structure) was as follows:

$$\begin{array}{l}{{\text{maximal}}}\_\mathrm{model}={\text{accuracy}}\sim 1+{\text{production}}*{\text{language}}+\\ \left(1+\mathrm{production + language }\left|\mathrm{ participant}\right.\right)+\\ \left(1+{\text{production}}\left|{\text{word}}\right.\right)\end{array}$$

For each Experiment, the maximal model capable of converging (which was then submitted to the mixed() function) included intercepts for participants and words:

$$\begin{array}{ll} \mathrm{final}\_ \mathrm{model}= \mathrm{accuracy}\sim 1 + \mathrm{production} + \mathrm{language}+ \mathrm{production}: \mathrm{language}+ \\ (1 | \mathrm{participant}) + (1 |\mathrm{word}) \end{array}$$

Model summaries and likelihood ratio test results for each Experiment are reported in Tables 6, 7, 8 and 9 below.

**Table 6:** Model summary: Experiment 1

Fixed effect	*Estimate*	*SE*	*z*	*P(z)*
Intercept	0.5453	0.1066	5.1138	<0.001
Production	1.1929	0.1046	11.4022	<0.001
Language	0.1786	0.1041	1.7155	0.086
Production x Language	0.5325	0.1599	3.3300	0.001
Random effect	*Variance*	*SD*		
Word (intercept)	0.1432	0.3784		
Participant (intercept)	0.3429	0.5856		

*SE* = standard error, *SD* = standard deviation

**Table 7:** Likelihood ratio tests: Experiment 1

Effect	*df*	*Chi squared*	*p*
Production	1	365.25	<.001
Language	1	21.64	<.001
Production × Language	1	11.08	<.001

**Table 8:** Model summary: Experiment 2

Fixed effect	*Estimate*	*SE*	*z*	*P(z)*
Intercept	0.2637	0.1082	2.4370	0.015
Production	1.7724	0.1117	15.8637	<0.001
Language	0.1159	0.1084	1.0687	0.285
Production × Language	0.2038	0.1622	1.2566	0.209
Random effect	*Variance*	*SD*		
Word (intercept)	0.2104	0.4587		
Participant (intercept)	0.3309	0.5752		

*SE* = standard error, *SD* = standard deviation

**Table 9:** Likelihood ratio tests: Experiment 2

Effect	*df*	*Chi squared*	*p*
Production	1	607.93	<.001
Language	1	4.61	.032
Production × Language	1	1.57	.211

## Appendix 2

### Exploratory hierarchical regression analyses of the L2 production effect

Exploratory hierarchical regression analyses were performed on the L2 (second language) production effect magnitude for each individual. L2 production effect scores for each individual were calculated by subtracting the “Silent” condition dprime score from the “Spoken” condition dprime score, both in the L2 only (Tables 10, 11 and 12). Difference scores were also calculated by subtracting L1 (first language) production effect magnitude (L1 Spoken minus L1 Silent) from the L2 production effect scores for each individual (Tables 13, 14 and 15). Regression analyses were conducted on each of the above scores separately for each Experiment (Tables 10, 11, 13, and 14) and for the two Experiments combined (Tables 12 and 15). Predictor variables included: (1) age of acquisition, years of study, and number of other languages spoken (block 1); (2) self-rated L2 speaking, understanding, reading, and writing and level (block 2); and (3) L2 LexTALE score and L1 LexTALE score (block 3). All continuous variables were zero-centered. Categorical variables were dummy-coded: “Number of other languages” (zero, one, two, or three other languages) was coded with reference to “zero” as Variable 1 (one = 1, otherwise = 0), Variable 2 (two = 1, otherwise = 0) and Variable 3 (three = 1, otherwise = 0), and “Self-rated L2 level” (A2, B1, B2, or C1) was coded with reference to “A2” as Variable 1 (B1 = 1, otherwise = 0 ), Variable 2 (B2 = 1, otherwise = 0), and Variable 3 (C1 = 1, otherwise = 0).

The regression models did not account for statistically significant proportions of the L2 production effect variance, and adding successive blocks of predictors did not significantly improve the models (Tables 10, 11, and 12). Only the first block of predictors in Experiment 2 accounted for a statistically significant proportion of the L2 minus L1 production effect variance, though adding successive blocks of predictors did not significantly improve the model (Table 14). In Experiment 1, speaking one additional language and self-rated L2 writing ability showed statistically significant negative and positive relationships, respectively, with L2 production effect magnitude (Table 10). In Experiment 2, speaking three additional languages showed a statistically significant positive relationship with L2 production effect magnitude (Table 11) and with L2 minus L1 production effect magnitude (Table 14), although this result should be interpreted with caution because only two participants in Experiment 2 spoke three additional languages.

**Table 10:** Experiment 1: Hierarchical regression analysis of the L2 production effect

	Regression 1		Regression 2		Regression 3	
Predictor	*Estimate (SE)*	*p*	*Estimate (SE)*	*p*	*Estimate (SE)*	*p*
Intercept	2.86 (0.59)	<.001	5.78 (1.71)	.001	5.60 (1.77)	.003
L2 Age	-0.02 (0.14)	.889	-0.13 (0.14)	.375	-0.11 (0.14)	.460
L2 Years	0.04 (0.13)	.735	-0.06 (0.13)	.666	-0.05 (0.14)	.700
Other languages (0, 1, 2, or 3)						
1 other language (versus 0)	-1.50 (0.69)	.035	-1.65 (0.77)	.037	-1.62 (0.79)	.047
2 other languages (versus 0)	-0.91 (0.69)	.193	-1.23 (0.80)	.129	-1.18 (0.82)	.159
3 other languages (versus 0)	-2.03 (1.04)	.057	-1.76 (1.06)	.103	-1.69 (1.09)	.130
L2 Speak			-0.27 (0.32)	.413	-0.27 (0.33)	.422
L2 Understand			0.30 (0.35)	.395	0.29 (0.36)	.430
L2 Read			-0.62 (0.35)	.084	-0.55 (0.37)	.139
L2 Write			0.78 (0.36)	.036	0.79 (0.37)	.036
L2 Level (A2, B1, B2, C1)						
Level B1 (versus A2)			-2.90 (2.00)	.154	-2.81 (2.07)	.181
Level B2 (versus A2)			-3.05 (1.83)	.102	-2.93 (1.90)	.131
Level C1 (versus A2)			-1.96 (1.96)	.323	-1.68 (2.05)	.419
LexTALE L2					-2.19 (3.16)	.491
LexTALE L1					2.45 (5.25)	.643
R2	0.11	.313	0.31	.103	0.32	.178
R2 change	0.11	.272	0.21	.102	0.01	.760

L2 Age = the age at which participants reported to have started to learn the L2, L2 Years = the number of years participants reported to have studied the L2, Other Languages = the number of other languages participants reported to speak (zero other languages *n* = 8; one other language *n* = 24; two other languages *n* = 22; three other languages *n* = 4), L2 Speak = self-rated L2 speaking ability on a scale of 1 to 7 (1 = not at all, 7 = fluent), L2 Understand = Self-rated L2 understanding ability (1 = not at all, 7 = fluent), L2 Read = self-rated L2 reading ability (1 = not at all, 7 = fluent), L2 Write = Self-rated L2 writing ability (1 = not at all, 7 = fluent), L2 Level = Self-reported L2 level (A2, B1, B2, or C1), LexTALE = Percent correct performance on the LexTALE lexical decision task, *SE* = standard error

All of the above predictor variable labels and other abbreviations apply to Tables 11, 12, 13, 14 and 15 below.

**Table 11:** Experiment 2: Hierarchical regression analysis of the L2 production effect

	Regression 1		Regression 2		Regression 3	
Predictor	*Estimate (SE)*	*p*	*Estimate (SE)*	*P*	*Estimate (SE)*	*p*
Intercept	1.89 (0.32)	<.001	2.05 (1.24)	.104	2.36 (1.26)	.067
L2 Age	0.02 (0.03)	.553	0.02 (0.04)	.557	0.02 (0.04)	.559
L2 Years	0.02 (0.03)	.478	0.001 (0.04)	.962	-0.01 (0.04)	.893
Other languages (0, 1, 2, or 3)						
1 other language (versus 0)	0.05 (0.54)	.937	0.10 (0.57)	.867	0.17 (0.57)	.767
2 other languages (versus 0)	-0.37 (0.61)	.552	-0.40 (0.67)	.552	-0.50 (0.67)	.458
3 other languages (versus 0)	3.00 (1.39)	.035	2.91 (1.46)	.052	3.25 (1.48)	.034
L2 Speak			-0.13 (0.46)	.777	-0.07 (0.47)	.887
L2 Understand			0.14 (0.39)	.732	0.16 (0.40)	.681
L2 Read			0.12 (0.36)	.738	0.14 (0.36)	.709
L2 Write			0.12 (0.33)	.730	0.16 (0.34)	.629
L2 Level (A2, B1, B2, C1)						
Level B1 (versus A2)			-0.62 (1.03)	.553	-0.96 (1.06)	.373
Level B2 (versus A2)			-0.37 (1.36)	.787	-0.59 (1.38)	.670
Level C1 (versus A2)			0.25 (1.71)	.884	-0.26 (1.75)	.884
LexTALE L2					-0.01 (0.03)	.763
LexTALE L1					0.05 (0.04)	.169
R2	0.14	.149	0.23	.384	0.27	.399
R2 change	0.14	.170	0.09	.661	0.03	.382

Other Languages: zero other languages *n* = 29; one other language *n* = 15; two other languages *n* = 11; three other languages *n* = 2

**Table 12:** Experiments 1 and 2: Hierarchical regression analysis of the L2 production effect

	Regression 1		Regression 2		Regression 3	
Predictor	*Estimate (SE)*	*p*	*Estimate (SE)*	*p*	*Estimate (SE)*	*p*
Intercept	2.13 (0.28)	<.001	2.92 (0.86)	<.001	3.03 (0.87)	<.001
L2 Age	0.02 (0.03)	.632	0.01 (0.03)	.765	0.01 (0.03)	.785
L2 Years	0.05 (0.03)	.120	0.02 (0.03)	.511	0.02 (0.04)	.619
Other languages (0, 1, 2, or 3)						
1 other language (versus 0)	-0.53 (0.40)	.181	-0.46 (0.40)	.249	-0.44 (0.40)	.271
2 other languages (versus 0)	-0.34 (0.41)	.412	-0.39 (0.43)	.368	-0.42 (0.44)	.338
3 other languages (versus 0)	-0.11 (0.79)	.894	-0.03 (0.78)	.968	0.01 (0.79)	.986
L2 Speak			-0.10 (0.24)	.685	-0.08 (0.25)	.754
L2 Understand			0.25 (0.25)	.330	0.25 (0.25)	.319
L2 Read			-0.22 (0.24)	.360	-0.21 (0.24)	.387
L2 Write			0.33 (0.22)	.137	0.32 (0.22)	.152
L2 Level (A2, B1, B2, C1)						
Level B1 (versus A2)			-1.23 (0.86)	.156	-1.37 (0.87)	.118
Level B2 (versus A2)			-1.01 (0.89)	.260	-1.10 (0.90)	.227
Level C1 (versus A2)			-0.36 (1.08)	.740	-0.52 (1.10)	.636
LexTALE L2					<0.01 (0.03)	.998
LexTALE L1					0.04 (0.04)	.303
R2	0.04	.499	0.13	.230	0.14	.298
R2 change	0.04	.483	0.09	.153	0.01	.581

**Table 13:** Experiments 1: Hierarchical regression analysis of the L2 minus L1 production effect

	Regression 1		Regression 2		Regression 3	
Predictor	*Estimate (SE)*	*p*	*Estimate (SE)*	*p*	*Estimate (SE)*	*p*
Intercept	2.05 (0.62)	.002	4.32 (1.95)	.032	4.49 (2.02)	.032
L2 Age	0.08 (0.15)	.574	0.01 (0.16)	.942	0.03 (0.17)	.861
L2 Years	0.14 (0.13)	.287	0.08 (0.15)	.601	0.09 (0.16)	.563
Other languages (0, 1, 2, or 3)						
1 other language (versus 0)	-1.46 (0.72)	.049	-1.32 (0.88)	.138	-1.20 (0.91)	.193
2 other languages (versus 0)	-1.14 (0.72)	.120	-1.03 (0.91)	.264	-0.92 (0.94)	.332
3 other languages (versus 0)	-1.89 (1.09)	.088	-1.53 (1.21)	.212	-1.37 (1.25)	.277
L2 Speak			-0.18 (0.36)	.622	-0.15 (0.38)	.695
L2 Understand			0.15 (0.40)	.703	0.22 (0.42)	.601
L2 Read			-0.35 (0.40)	.385	-0.37 (0.42)	.383
L2 Write			0.32 (0.41)	.435	0.32 (0.42)	.448
L2 Level (A2, B1, B2, C1)						
Level B1 (versus A2)			-2.51 (2.28)	.278	-2.82 (2.37)	.240
Level B2 (versus A2)			-2.62 (2.09)	.217	-2.91 (2.17)	.188
Level C1 (versus A2)			-1.82 (2.24)	.421	-2.04 (2.35)	.390
LexTALE L2					-1.36 (3.61)	.707
LexTALE L1					-2.96 (6.00)	.625
R2	0.09	.410	0.16	.726	0.17	.821
R2 change	0.09	.468	0.07	.812	0.01	.761

**Table 14:** Experiments 2: Hierarchical regression analysis of the L2 minus L1 production effect

	Regression 1		Regression 2		Regression 3	
Predictor	*Estimate (SE)*	*p*	*Estimate (SE)*	*p*	*Estimate (SE)*	*p*
Intercept	0.55 (0.33)	.103	0.92 (1.27)	.470	1.08 (1.31)	.414
L2 Age	0.02 (0.03)	.607	0.05 (0.04)	.234	0.04 (0.04)	.268
L2 Years	0.05 (0.04)	.175	0.03 (0.04)	.403	0.03 (0.04)	.427
Other languages (0, 1, 2, or 3)						
1 other language (versus 0)	-0.18 (0.56)	.748	-0.18 (0.58)	.761	-0.11 (0.60)	.856
2 other languages (versus 0)	-0.70 (0.64)	.280	-0.96 (0.68)	.168	-1.00 (0.70)	.158
3 other languages (versus 0)	3.37 (1.45)	.024	3.09 (1.50)	.045	3.28 (1.54)	.039
L2 Speak			0.45 (0.47)	.342	0.54 (0.49)	.277
L2 Understand			-0.14 (0.40)	.735	-0.15 (0.41)	.722
L2 Read			0.60 (0.37)	.107	0.62 (0.37)	.103
L2 Write			-0.25 (0.34)	.472	-0.21 (0.35)	.558
L2 Level (A2, B1, B2, C1)						
Level B1 (versus A2)			0.40 (1.06)	.709	0.25 (1.10)	.824
Level B2 (versus A2)			-0.67 (1.40)	.636	-0.86 (1.44)	.553
Level C1 (versus A2)			-0.49 (1.76)	.780	-0.71 (1.82)	.699
LexTALE L2					-0.02 (0.03)	.490
LexTALE L1					0.02 (0.04)	.581
R2	0.22	.024	0.32	.085	0.34	.147
R2 change	0.22	.030	0.11	.478	0.01	.706

**Table 15:** Experiments 1 and 2: Hierarchical regression analysis of the L2 minus L1 production effect

	Regression 1		Regression 2		Regression 3	
Predictor	*Estimate (SE)*	*p*	*Estimate (SE)*	*p*	*Estimate (SE)*	*p*
Intercept	0.90 (0.30)	.003	0.78 (0.94)	.405	0.80 (0.96)	.405
L2 Age	0.02 (0.03)	.648	0.03 (0.04)	.503	0.02 (0.04)	.516
L2 Years	0.08 (0.03)	.027	0.06 (0.04)	.125	0.06 (0.04)	.140
Other languages (0, 1, 2, or 3)						
1 other language (versus 0)	-0.37 (0.42)	.381	-0.40 (0.44)	.355	-0.40 (0.44)	.365
2 other languages (versus 0)	-0.37 (0.44)	.398	-0.46 (0.48)	.331	-0.47 (0.48)	.332
3 other languages (versus 0)	0.39 (0.83)	.639	0.31 (0.86)	.718	0.31 (0.87)	.714
L2 Speak			0.01 (0.27)	.972	0.01 (0.27)	.961
L2 Understand			0.06 (0.28)	.823	0.06 (0.28)	.822
L2 Read			0.13 (0.26)	.617	0.13 (0.27)	.615
L2 Write			0.01 (0.24)	.956	0.01 (0.24)	.956
L2 Level (A2, B1, B2, C1)						
Level B1 (versus A2)			0.03 (0.94)	.972	0.01 (0.96)	.989
Level B2 (versus A2)			0.07 (0.98)	.939	0.06 (0.99)	.949
Level C1 (versus A2)			0.41 (1.19)	.731	0.39 (1.21)	.749
LexTALE L2					-0.001 (0.03)	.966
LexTALE L1					0.01 (0.04)	.892
R2	0.07	.155	0.10	.540	0.10	.702
R2 change	0.07	.181	0.03	.887	<0.001	.991

## Footnotes

1 A misspelling of one German word (President rather than Präsident) was corrected approximately halfway through Experiment 2 and was also removed from all analyses. There were also three German words that were plural versions of other words in the pool (e.g., “Menschen”, plural of “Mensch”). All results in Experiments 1 and 2 remained unchanged when these words were removed from the analyses.

2 Trials where participants correctly spoke aloud were included even if pronunciation was unclear or contained errors. We reasoned that pronunciation errors could lead to recognition errors, which should reduce the predicted production effect.

3 Additionally, a subset of participants in Experiment 2 (*n* = 51) were rated by bilingual coders as having overall “good,” “ok,” or “poor” L1 and L2 pronunciation. Visual examination showed that “good” L2 pronouncers (*n* = 34) showed a slightly numerically larger L2 production effect than “ok” L2 pronouncers (*n* = 15). Only two participants received a “poor” rating, and did so in both their L2 and their L1. Although the ratings are crude, they suggest that including a variety of pronunciations may indeed have decreased the average size of the L2 production effect.

## Code availability

The custom R code used for the current study are freely available on psycharchives.org.
